# Fatal pseudoaneurysm of the subclavian artery secondary to infected modified Blalock–Taussig–Thomas shunt: a case report

**DOI:** 10.1093/jscr/rjaf1086

**Published:** 2026-01-20

**Authors:** Mourad Boukheloua, Yehya Khlidj, Aziza Baali, Selma Aroua, Mohamed Berrehal, Mohamed Rifai

**Affiliations:** Faculty of Medicine, University of Health Sciences, Moudjahid Doctor Youcef El Khatib, Road 8 Lieutenant Mohamed Benarfa El Biar 16000, Algiers, Algeria; Cardiology Department, Hussein-Dey Nafissa Hammoud University Hospital Center, Road 94 Boudjemaa Moghni, Hussein-Dey 16005, Algiers, Algeria; Department of Physiology and Molecular Biology, University of Sciences and Technology Houari Boumediene, BP 32 Bab Ezzouar 16111, Algiers, Algeria; Faculty of Medicine, University of Health Sciences, Moudjahid Doctor Youcef El Khatib, Road 8 Lieutenant Mohamed Benarfa El Biar 16000, Algiers, Algeria; Department of Physiology and Molecular Biology, University of Sciences and Technology Houari Boumediene, BP 32 Bab Ezzouar 16111, Algiers, Algeria; Medical Imaging and Radiology Department, Zeralda Burns Center, Zeralda 16063, Algiers, Algeria; Faculty of Medicine, University of Health Sciences, Moudjahid Doctor Youcef El Khatib, Road 8 Lieutenant Mohamed Benarfa El Biar 16000, Algiers, Algeria; Cardiology Department, Hussein-Dey Nafissa Hammoud University Hospital Center, Road 94 Boudjemaa Moghni, Hussein-Dey 16005, Algiers, Algeria; Faculty of Medicine, University of Health Sciences, Moudjahid Doctor Youcef El Khatib, Road 8 Lieutenant Mohamed Benarfa El Biar 16000, Algiers, Algeria; Cardiology Department, Hussein-Dey Nafissa Hammoud University Hospital Center, Road 94 Boudjemaa Moghni, Hussein-Dey 16005, Algiers, Algeria; Faculty of Medicine, Menoufia University, Gamal Abd El Nasr st Shebin El-Koum 32511 Menoufia, Egypt

**Keywords:** modified Blalock–Taussig–Thomas shunt, pseudoaneurysm, subclavian artery, congenital heart disease, pediatric cardiac surgery

## Abstract

Pseudoaneurysm is a rare but life-threatening complication of the modified Blalock–Taussig–Thomas shunt (mBTTS). We report a 3-year-old girl with pulmonary atresia and ventricular septal defect who underwent staged bilateral mBTTS and presented three months later with fever, acute respiratory distress, and worsening cyanosis. Initial findings suggested severe pneumonia, with right lower-lobe consolidation and abnormal mediastinal contour on chest radiography. Echocardiography and computed tomography angiography revealed a massive, thrombosed pseudoaneurysm of the right subclavian artery at the mBTTS anastomosis, complete thrombosis of the right pulmonary artery, right lung collapse, and concomitant pneumonia. Additional findings included left isomerism and a horseshoe kidney. Despite urgent broad-spectrum antibiotics and referral for emergency surgery, the child rapidly deteriorated and died before intervention. This case emphasizes the need for high clinical suspicion and early advanced imaging in post-mBTTS patients presenting with sepsis or cardiorespiratory compromise.

## Introduction

The modified Blalock–Taussig–Thomas shunt (mBTTS) is a standard palliative procedure for cyanotic congenital heart disease with reduced pulmonary blood flow when primary repair is not feasible [1–3]. By creating a systemic-to-pulmonary connection, it augments pulmonary blood flow, improves oxygenation, and stabilizes patients until they are candidates for definitive surgery.

Although usually safe, mBTTS is associated with early and late complications, including shunt stenosis or occlusion, seroma, and pseudoaneurysm formation [4, 5]. Pseudoaneurysm is particularly rare but carries a high risk of rupture, hemorrhage, thromboembolism, and compression of adjacent structures [6].

We report a fatal infected pseudoaneurysm of the subclavian artery shortly after bilateral mBTTS in a 3-year-old child, emphasizing the need for high clinical suspicion and early imaging in deteriorating post-shunt patients.

## Case report

A 3-year-old girl with pulmonary atresia, large conotruncal ventricular septal defect with right-to-left shunt and patent ductus arteriosus was diagnosed in the neonatal period. Three months before presentation she underwent staged bilateral mBTTS. A 6-mm polytetrafluoroethylene graft was anastomosed between the right subclavian and right pulmonary arteries with unifocalization plasty, followed by a 5-mm left-sided graft between the left subclavian and left pulmonary arteries. The peri-operative course was uneventful and she was discharged in good condition.

She presented to the emergency department with 3 days of fever, progressive respiratory distress, and worsening cyanosis. On arrival, she was acutely ill: temperature 39.2°C, heart rate 155 beats/min, respiratory rate 48 breaths/min, oxygen saturation 78% on room air, and blood pressure 85/50 mmHg. She had central and peripheral cyanosis, intercostal retractions, and decreased breath sounds over the right lower lung field. A continuous murmur compatible with shunt flow was audible but softer than on previous examinations.

**Figure 1:**
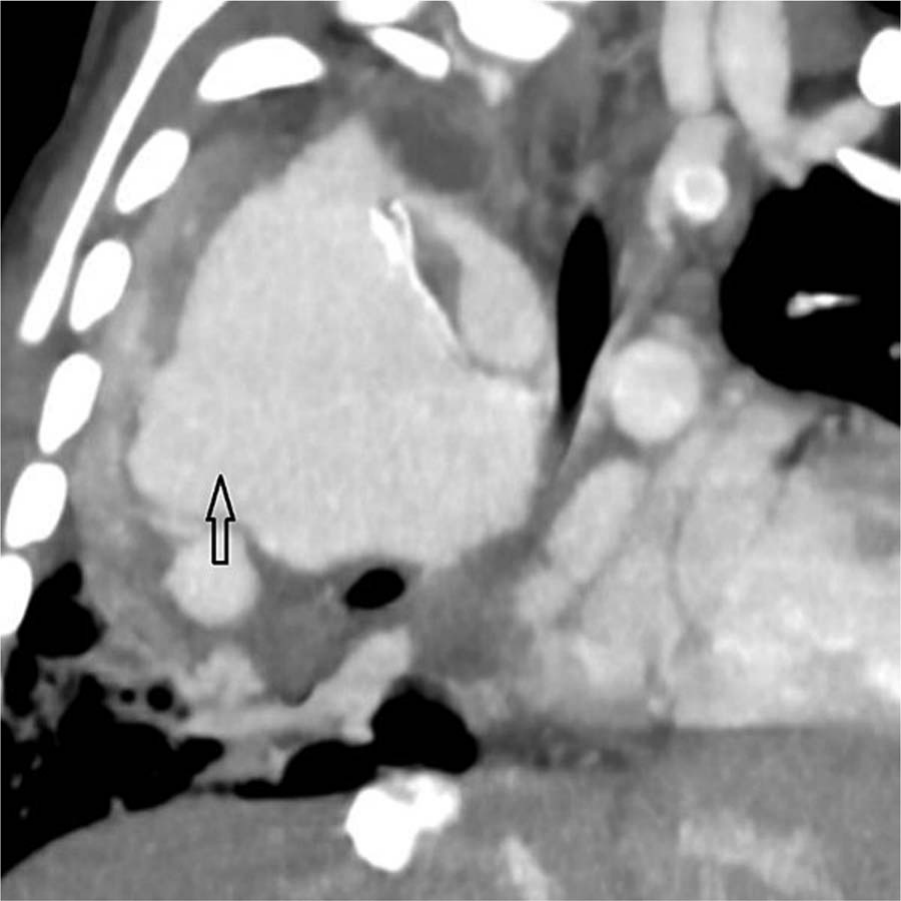
Coronal oblique maximum-intensity-projection reconstruction of thoracic CT angiography showing a large thrombosed pseudoaneurysm of the right subclavian artery at the anastomosis with the modified Blalock–Taussig–Thomas shunt, causing significant mediastinal mass effect.

**Figure 2:**
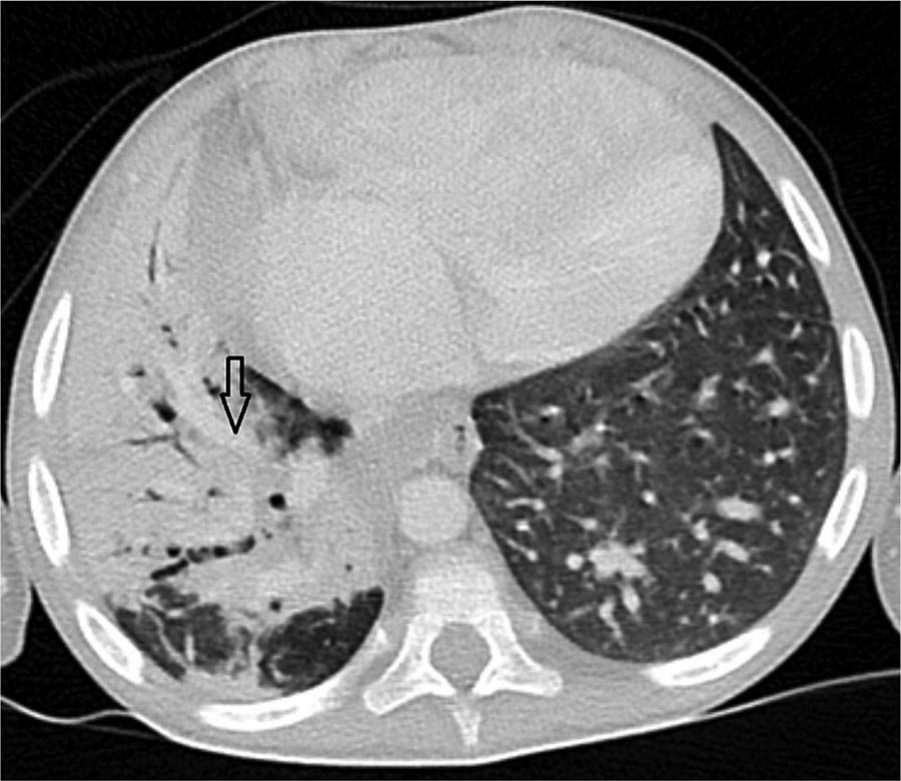
Axial CT angiogram in lung window demonstrating right lower-lobe consolidation and volume loss with mediastinal shift, consistent with pneumonia and compression by the pseudoaneurysm.

Laboratory tests showed leucocytosis (24 500/μL, 85% neutrophils), C-reactive protein 185 mg/L, and procalcitonin 4.2 ng/mL. Chest radiography revealed dense right lower-lobe opacity with abnormal mediastinal contour and rightward tracheal deviation.

**Figure 3:**
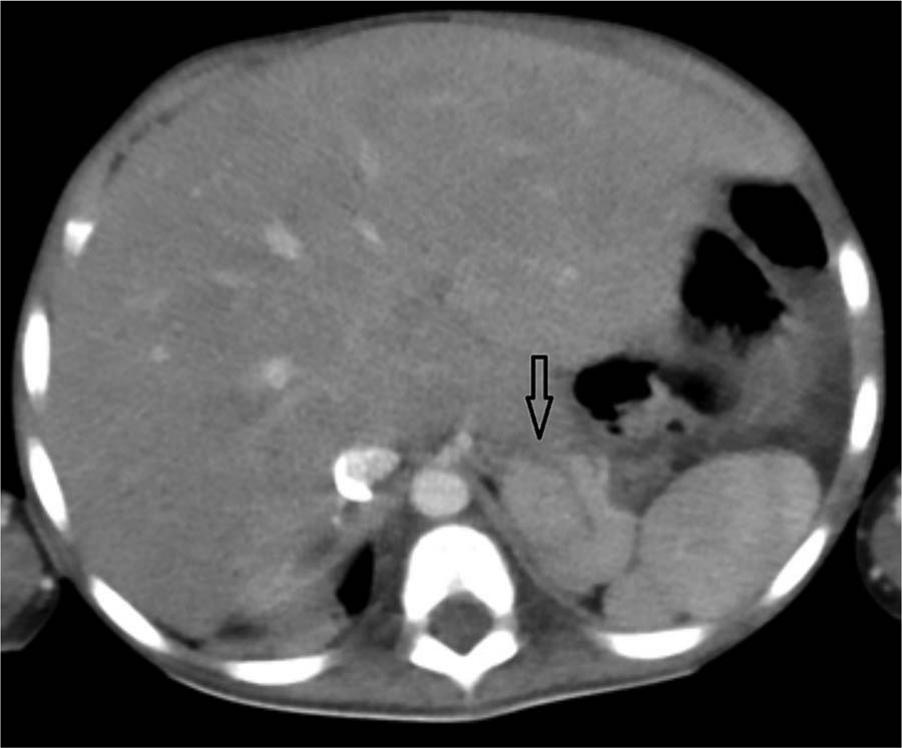
Axial abdominal CT showing central liver and polysplenism, compatible with left isomerism.

**Figure 4:**
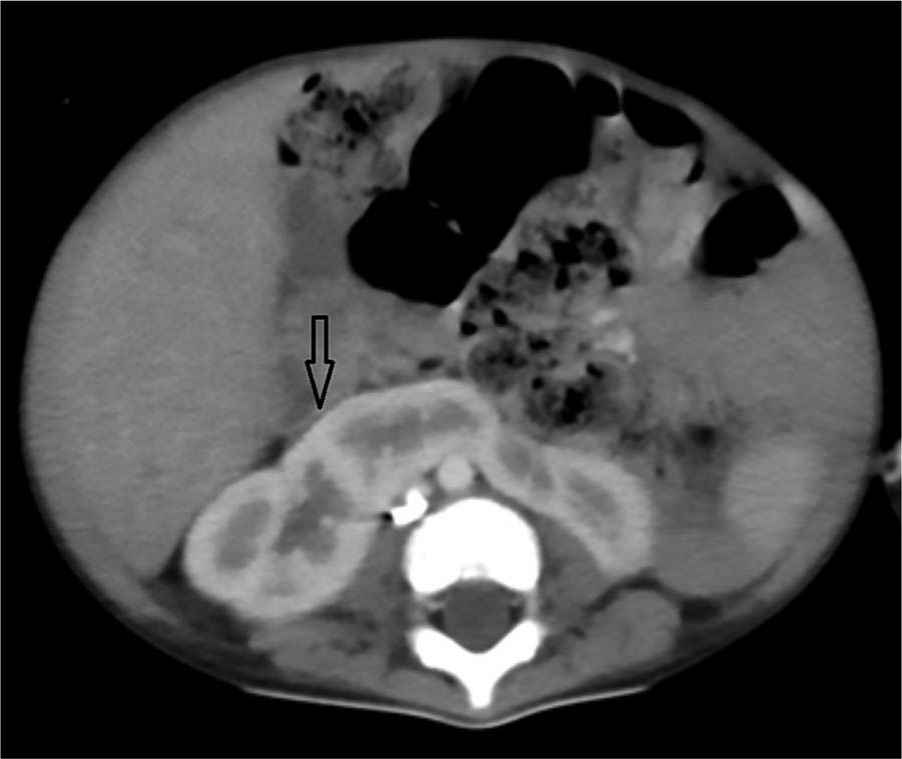
Axial abdominal CT demonstrating a horseshoe kidney.

Urgent transthoracic echocardiography demonstrated a large echodense mass in the right hemithorax, compression of the right atrium and reduced flow through the right-sided shunt. Computed tomography angiography (CTA) confirmed a massive saccular pseudoaneurysm (≈8 × 7 × 6 cm) arising from the anastomosis between the right subclavian artery and the mBTTS graft, completely thrombosed without active contrast extravasation (Fig. 1). There was complete thrombotic occlusion of the right pulmonary artery, right lung collapse due to extrinsic compression and right lower-lobe consolidation consistent with pneumonia (Fig. 2). CTA also revealed left isomerism with central liver and polysplenism (Fig. 3) and a horseshoe kidney (Fig. 4), suggesting a previously unrecognized polymalformative syndrome.

Broad-spectrum intravenous antibiotics (vancomycin and ceftazidime) were started for presumed infected shunt and pneumonia, and cardiothoracic surgeons were urgently consulted. Despite aggressive resuscitation, the child developed worsening hypoxaemia and hypotension and suffered cardiorespiratory arrest before surgical repair could be attempted. The family declined autopsy.

## Discussion

Pseudoaneurysm formation after mBTTS is rare but potentially catastrophic. It usually arises from disruption of the arterial wall at or near the anastomotic site, with blood contained by surrounding tissues to form a pulsatile haematoma that communicates with the arterial lumen [7]. Contributing factors include technical issues, mechanical stress, patient tissue fragility and—most importantly—infection [7]. In our patient, the close temporal relationship between systemic sepsis and imaging findings strongly suggests an infected pseudoaneurysm complicating an otherwise technically satisfactory shunt.

The clinical presentation of post-mBTTS pseudoaneurysm is heterogeneous, ranging from incidental discovery to life-threatening hemorrhage or cardiopulmonary collapse [8]. Symptoms may reflect compression (dyspnoea, dysphagia, superior vena cava obstruction), thrombotic complications (shunt occlusion, pulmonary embolism), or rupture [6–8]. In this case, the initial picture mimicked severe pneumonia, and the mediastinal contour abnormality seen on chest radiography could easily have been overlooked. This underscores a key diagnostic message: in children with complex congenital heart disease and prior shunt surgery, apparently straightforward respiratory infections may conceal serious vascular complications.

Literature suggests that subclavian artery anastomosis and grafts ≥4 mm are associated with lower rates of serious adverse events [6, 9]. Both features were present here, supporting infection as the principal driver of pseudoaneurysm formation. Preventive strategies therefore include meticulous surgical technique, strict peri-operative antibiotic prophylaxis, rapid treatment of postoperative infections, and education of caregivers about red-flag symptoms. Regular postoperative follow-up with clinical review and selective imaging may enable earlier detection [4, 6, 9].

Management of mBTTS-related pseudoaneurysm requires rapid haemodynamic stabilization, broad-spectrum antibiotics when infection is suspected and urgent surgical intervention to resect the pseudoaneurysm, remove infected material, and revise or replace the shunt [7, 9–11]. Endovascular techniques, such as covered stents or coil embolization, have been reported in selected cases but experience in small children is limited [12]. Outcome depends largely on timely recognition and the feasibility of prompt definitive repair.

Our case highlights several practical lessons: clinicians should maintain a high index of suspicion for pseudoaneurysm in any post-mBTTS patient with new cardiorespiratory instability or sepsis; standard chest radiography may be misleading, and early echocardiography and CTA are often decisive; and pseudoaneurysm in this setting must be treated as a surgical emergency given the risk of rapid progression, thrombosis, compression, and death. As a single case without histopathological confirmation, our report has inherent limitations, but it adds to the scarce pediatric literature and reinforces the need for vigilant surveillance and prompt imaging in this vulnerable population.

## Data Availability

Not applicable.
